# A Narrative Review of Zebrafish Models of Diabetes Mellitus

**DOI:** 10.7759/cureus.107992

**Published:** 2026-04-29

**Authors:** Indrani Sarma, Dibyajyoti Saikia

**Affiliations:** 1 Pharmacology, All India Institute of Medical Sciences, Guwahati, Guwahati, IND

**Keywords:** animal models, diabetes mellitus, drug discovery, drug screening, hyperglycemia, insulin resistance, metabolic syndrome, zebrafish, β-cell regeneration

## Abstract

Diabetes mellitus is a major chronic metabolic disease characterized by persistent hyperglycemia resulting from impaired insulin secretion, impaired insulin action, or both, and is associated with microvascular and macrovascular complications. Although rodent models remain central to diabetes research, zebrafish (*Danio rerio*) have emerged as a powerful complementary vertebrate model because of their genetic and physiological conservation with humans, rapid development, optical transparency during early life stages, high fecundity, low maintenance cost, and suitability for high-throughput drug screening. Several reviews have addressed specific aspects of zebrafish diabetes research, but fewer provide an integrated comparison across the major genetic, chemical, and dietary models while also emphasizing translational relevance, model selection, and practical limitations. This narrative review, therefore, provides a comparative overview of the major zebrafish diabetes models and their principal applications. We group models into genetic models with predominant beta-cell dysfunction or insulin deficiency and those with predominant insulin resistance or type 2 diabetes-like metabolic dysfunction; chemical models including beta-cell-toxic agents such as streptozotocin and alloxan, as well as metabolically oriented glucose immersion and endocrine-disruptor exposures; and dietary models based on overfeeding and high-fat, high-glucose, or combined cholesterol-glucose regimens. Across these model classes, we compare induction strategy, principal phenotype, relevance to type 1 versus type 2 diabetes, strengths, limitations, and typical research applications in drug discovery, regeneration, pharmacogenomics, and studies of complications. We also emphasize that there is no universally accepted single diagnostic glycemic threshold for diabetes in zebrafish; instead, model validation usually relies on sustained elevation of glucose relative to controls together with functional or mechanistic readouts such as glucose tolerance, insulin signaling, beta-cell mass, and lipid-metabolic changes. Despite important physiological differences from mammals and the strong regenerative capacity of zebrafish, the model provides a scalable vertebrate platform for mechanistic and early translational diabetes research.

## Introduction and background

Diabetes mellitus is a heterogeneous group of metabolic disorders characterized by chronic hyperglycemia caused by defects in insulin secretion, insulin action, or both. It is one of the major non-communicable diseases. The prevalence of diabetes has reached epidemic proportions, with type 2 diabetes mellitus (T2DM) contributing to about 90% of all diabetes cases. The International Diabetes Federation estimates that 589 million adults aged 20 to 79 years were living with diabetes in 2024, and that this number may rise to 853 million by 2050 [1]. Beyond abnormal glucose homeostasis, diabetes contributes to major microvascular and macrovascular complications, including retinopathy, nephropathy, neuropathy, myocardial infarction, stroke, and peripheral vascular disease [2]. Animals have long been used in the study of diabetes, both in terms of the understanding of the disease as well as in the research and development of therapeutic products. By far the most common model has been rodents, but a growing body of work suggests that vertebrate models other than rodents may also have important roles in particular areas of research related to diabetes. As a vertebrate model that is other than a rodent, the zebrafish, *Danio rerio*, is an emerging model system in diabetes research, and based on various characteristics of this animal, it can be an excellent model system for studying diabetes in a unique manner [3-5]. In fact, zebrafish are one of the unique model systems that, by virtue of certain special properties, could be optimally utilized for the study of metabolic disorders such as diabetes. Approximately 70% of human genes have at least one zebrafish orthologue, and key pathways involved in pancreatic development, glucose homeostasis, insulin signaling, and lipid metabolism are conserved between zebrafish and mammals [6]. They become transparent for a brief period at early embryonic stages, provide unlimited numbers of progeny upon breeding, mature, and organ functions mature rapidly with development being completed within five days post-fertilization, and they also possess a high degree of genetic as well as physiological homology to mammals.

The establishment of zebrafish models for diabetes has evolved considerably over the past two decades, with researchers developing diverse approaches to induce hyperglycemia and model both type 1 and type 2 diabetes [5-7]. These models have been successfully employed to investigate disease mechanisms, screen potential therapeutic compounds, study diabetic complications at the molecular and cellular levels, and identify novel biomarkers [2,3,8]. The conservation of key metabolic pathways, including glucose homeostasis, insulin signaling, and lipid metabolism, between zebrafish and mammals provides a strong foundation for translational research [9,10]. 

Several recent reviews have discussed zebrafish diabetes research, but many of them focus on specific subdomains, such as model construction, type 2 diabetes-oriented induction methods, preclinical utility, or diabetic complications [2,4-7]. Fewer reviews explicitly compare the main genetic, chemical, and dietary model classes in one framework while also distinguishing insulin-deficient from insulin-resistant states and discussing when each model is most appropriate for mechanistic studies, translational research, drug discovery, or complication work. The present article, therefore, aims to provide an integrated narrative overview of the major zebrafish diabetes models, with emphasis on comparative model evaluation, translational relevance, and the practical advantages and limitations of zebrafish relative to mammalian systems.

Approach to literature identification

This article is a narrative review. Literature was identified primarily through PubMed, Scopus, and Google Scholar using combinations of the terms “zebrafish”, “*Danio rerio*”, “diabetes”, “hyperglycemia”, “insulin resistance”, “beta-cell”, “streptozotocin”, “alloxan”, “high-fat diet”, and “drug screening”. English-language publications available up to March 2026 were considered. Selection included studies and reviews that described model induction, phenotype validation, translational relevance, complications, and representative applications. No risk-of-bias assessment or quantitative synthesis was performed.

## Review

Zebrafish as a model organism for diabetes research

Almost 70% of human genes have at least one orthologue of zebrafish, with conservation of key genes involved in glucose metabolism, insulin signaling, and pancreatic development [8]. The zebrafish pancreas develops early, with insulin-producing β-cells appearing by 24 hours post-fertilization (hpf) and glucagon-producing α-cells by 28 hpf [9]. The organization of pancreatic islets in zebrafish differs from that of mammals, with a principal islet and numerous secondary islets distributed throughout the pancreatic tissue. Still, the cellular composition and hormonal regulation show remarkable conservation [11]. Metabolic pathways preserved in zebrafish and similar to those in humans include glucose homeostasis, insulin signaling, lipid metabolism, and pancreatic development, among others [12-17].

Zebrafish offer several advantages over rodent animal models [18,19]. The zebrafish larvae are transparent in the first few weeks of development, which enables real-time visualization of pancreatic islets and vascular changes without invasive procedures [2]. Their small size and relatively low cost facilitate screening of multiple compounds or conditions in parallel, making zebrafish particularly attractive for hypothesis generation, early drug screening, phenotype-based discovery, and regeneration studies [8]. Rapid generation of genetic models is possible using modern gene editing tools like clustered regularly interspaced short palindromic repeats/CRISPR-associated protein 9 (CRISPR/Cas9) and transcription activator-like effector nucleases (TALENs) [2,3,12]. Zebrafish can be bred and sustained in a minimal space and require fewer resources in comparison with rodent models, making them low-maintenance animals that are very cost-effective for research [13,20]. Zebrafish develop over a very short time, which allows the visualization of the progress of a disease as well as their response to drugs over a significantly shorter amount of time [9,10]. Zebrafish embryos and early larvae are not subject to the same ethical restrictions as mammalian models, making them ideal for various forms of research [20].

Zebrafish diabetes models can be broadly viewed in two mechanistic groups: models with predominant beta-cell dysfunction or insulin deficiency, and models with predominant insulin resistance or type 2 diabetes-like metabolic dysfunction. This distinction is useful because hyperglycemia alone does not capture the biological diversity of zebrafish diabetes models. Some protocols are designed mainly to ablate beta-cells or reduce insulin production, whereas others induce metabolic stress with preserved or even compensatory insulin output in early stages.

There is no universally accepted single diagnostic glycemic threshold for diabetes in zebrafish that applies across all developmental stages, tissues, and assay formats. In practice, studies usually validate a diabetic or diabetogenic phenotype through a combination of readouts: sustained elevation of blood, fasting, or whole-body glucose relative to controls; abnormal glucose tolerance testing where available; altered insulin content or insulin signaling; reduced beta-cell mass in insulin-deficient models; and associated metabolic changes such as hepatic steatosis, dyslipidemia, inflammation, or oxidative stress [6]. For this reason, model selection should be guided by the biological question rather than by glucose elevation alone.

Models of diabetes

Genetic Models

**Figure 1:**
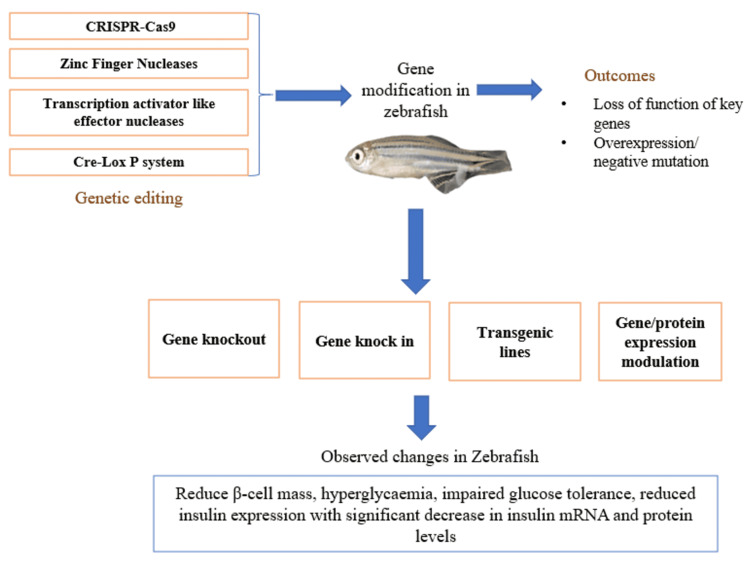
Genetic models of diabetes in zebrafish Gene editing and transgenic approaches in adult zebrafish disrupt key metabolic genes and pathways, producing diabetes-like changes including β-cell dysfunction, hyperglycemia, impaired glucose tolerance, reduced insulin expression, lipid accumulation, inflammation, and oxidative stress. The image of the zebrafish has been taken from our lab. The rest of the image is made by the authors using Microsoft PowerPoint (Microsoft® Corp., Redmond, WA).

Genetic models provide precise tools for investigating specific molecular mechanisms underlying diabetes pathogenesis. These models are typically used for study on type 1 diabetes mellitus (T1DM) (Figure 1) [2,12].

Predominant beta-cell dysfunction or insulin deficiency: Genetic models are particularly useful for probing causal mechanisms. Among the best characterized is the pancreatic and duodenal homeobox 1 (PDX1) mutant model. PDX1 is a transcription factor essential for pancreatic development and beta-cell function, and pathogenic variation in human PDX1 is associated with monogenic diabetes. In zebrafish, PDX1 mutation leads to pancreatic hypoplasia, reduced beta-cell mass, impaired glucose tolerance, reduced insulin expression, and hyperglycemia [16]. Because the phenotype is mechanistically anchored in beta-cell dysfunction, this model is especially valuable for studying pancreatic development, beta-cell maintenance, nutrient stress, and antidiabetic rescue strategies.

Defects in glucose sensing and insulin secretory pathways also fall within this broader mechanistic class. GLUT2 deficiency models disrupt glucose sensing in beta-cells and other metabolic tissues, resulting in impaired insulin secretion and disordered glucose metabolism [2,12]. These models are informative when the research focus is glucose sensing, beta-cell responsiveness, or early endocrine dysregulation rather than obesity-associated insulin resistance.

Predominant insulin resistance or type 2 diabetes-like metabolic dysfunction: Other genetic manipulations more closely reflect insulin resistance or broader metabolic dysfunction. GLUT12 mutants provide insight into peripheral glucose disposal, while insulin receptor (INSR) and downstream insulin signaling mutants model severe insulin resistance, abnormal growth, and altered lipid handling [2,12]. Similarly, mutations affecting IRS-PI3K-AKT signaling, Gpr27, DIO2, or related regulators can generate glucose intolerance, altered feeding behavior, reduced metabolic flexibility, or insulin resistance-like phenotypes [2,14].

Not all genetically altered lines are equally suitable as primary diabetes models. For example, glyoxalase 1 (glo1) knockout fish are especially useful for studying methylglyoxal accumulation, advanced glycation, oxidative stress, and vascular dysfunction, and may therefore be best viewed as mechanistically informative models for diabetic injury or complications rather than stand-alone models of classic diabetes onset [2,20]. Likewise, therapeutic silencing of centromere protein X (CENP-X) has been explored more as a modifier of hyperglycemia than as a foundational disease-induction model [14].

Taken together, genetic models offer the highest mechanistic precision and are attractive when the aim is to dissect causal pathways, identify modifiers, or test targeted rescue strategies. Their main strengths are reproducibility, biological specificity, and compatibility with imaging and molecular profiling. Their main limitations are that some lines have developmental phenotypes, compensatory adaptations, or disease trajectories that do not fully resemble adult-onset human diabetes. Accordingly, genetic models are strongest for mechanistic studies and pathway validation, and are often best complemented by chemical or dietary models when broader metabolic syndrome-like phenotypes are required.

PDX1 Mutant Models

The PDX1 gene encodes a transcription factor essential for pancreatic development and β-cell function. In humans, this mutation causes maturity-onset diabetes of the young (MODY4) [17]. The zebrafish PDX1 gene shows high similarity with mammalian genes and thus can be used to study this disease in more detail. In the PDX1 mutant zebrafish model developed by Zang et al. in 2015 using zinc-finger nuclease technology, a frameshift mutation resulted in loss of functional PDX1 protein [17]. The mutation exhibited pancreatic hypoplasia with reduced β-cell mass, hyperglycemia, impaired glucose tolerance, reduced insulin expression with significant decrease in insulin mRNA and protein levels. This model also showed downregulation of β-cell markers, including insulin, glut2, and neurod1, as well as response to anti-glycemic treatments, including metformin and insulin, validating its utility for drug screening. The animals also showed impaired pancreatic bud formation and reduced endocrine cell differentiation during embryonic development. This model has been used extensively, establishing the role of PDX1 in β-cell maintenance, gene interactions in diabetes pathogenesis, and compound screening that promote and enhance the β-cell function and regeneration.

Glucose Transporter Mutants

Glucose transporter 2 (GLUT2) deficiency models: GLUT2 (encoded by solute carrier family 2 member 2 (slc2a2) in zebrafish) plays a critical role in glucose sensing and insulin secretion [2,9]. GLUT2 knockout zebrafish exhibit impaired glucose sensing, altered insulin secretion, hyperglycemia, and metabolic dysfunction. Studies on these mutants have revealed the role of GLUT2 in the glucose-sensing mechanism of β-cells as well as tissue-specific effects of GLUT2 deficiency on glucose metabolism [3,21,22].

Glucose transporter 12 (GLUT12) deficiency models: GLUT12 is an insulin-responsive glucose transporter expressed in skeletal muscle and adipose tissue. Zebrafish GLUT12 mutants provide insights into peripheral insulin resistance [3].

Insulin Signaling Pathway Mutants

INSR mutants: INSR mutations cause severe insulin resistance syndromes in humans. Models in zebrafish have been generated to mimic that effect [3]. These models show severe insulin resistance, hyperglycemia despite hyperinsulinemia, growth retardation and developmental delays, and altered lipid metabolism with increased adiposity.

IRS and downstream signaling mutants: INSR substrate (IRS) proteins, downstream signaling components (phosphoinositide 3-kinase (PI3K), protein kinase B (AKT) mutations have been studied and have been found to develop progressive insulin resistance [15].

Other Genetic Models

glo1 knockout model: The glo1 gene encodes an enzyme that detoxifies methylglyoxal, a reactive dicarbonyl compound elevated in diabetes. The glo1 knockout zebrafish model exhibits accumulation of advanced glycation end products (AGEs), oxidative stress, inflammation, and vascular dysfunction [3].

Deiodinase type 2 (DIO2) knockout model: DIO2 regulates thyroid hormone metabolism, which influences glucose homeostasis. DIO2 gene knockout in zebrafish shows an altered glucose metabolism, reduced metabolic rate, and impaired thermogenesis [3].

G protein-coupled receptor 27 (Gpr27) knockout model: Gpr27 knockout in zebrafish shows insulin resistance, glucose intolerance, and altered feeding behavior [3].

CENP-X silencing model: A novel therapeutic target, CENP-X, has been shown to ameliorate hyperglycemia in zebrafish models [15].

Chemical induction models

**Figure 2:**
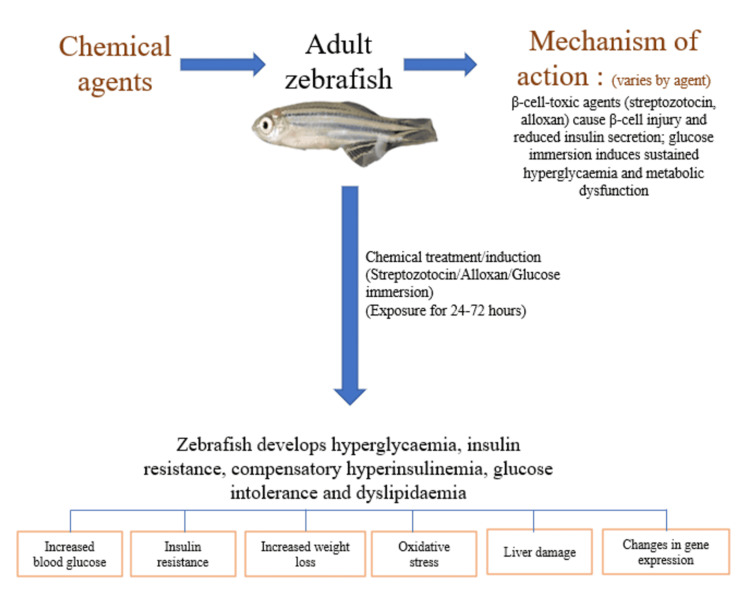
Schematic representation of chemically induced diabetes models in adult zebrafish Chemical induction of diabetes-like states in adult zebrafish can be achieved using β-cell-toxic agents such as streptozotocin and alloxan, or by metabolically oriented approaches such as glucose immersion. β-cell-toxic agents primarily cause β-cell injury, reduced insulin secretion, and hyperglycemia, thereby modelling insulin-deficient states. In contrast, glucose immersion mainly induces sustained hyperglycemia, glucose intolerance, and insulin-resistant or metabolic dysfunction-like features, sometimes with compensatory hyperinsulinemia. Depending on the agent, dose, and duration of exposure, these models may also show oxidative stress, altered gene expression, weight loss, dyslipidemia, and liver injury. The image of the zebrafish was taken from our lab. The rest of the image is made by the authors using Microsoft PowerPoint (Microsoft® Corp., Redmond, WA).

Chemical induction provides rapid, cost-effective methods to generate diabetic phenotypes in zebrafish (Figure 2). Chemical induction models offer a rapid and relatively economical route to zebrafish hyperglycemia. Mechanistically, they can be divided into beta-cell-toxic models, which primarily generate insulin-deficient states, and metabolic-stress models, which are more useful for studying insulin resistance-like or diabetogenic phenotypes.

These models are particularly useful for acute studies, drug screening, and investigating specific aspects of diabetes pathophysiology [8,22,23].

Beta-Cell-Toxic Models: Streptozotocin (STZ) and Alloxan

STZ is a glucosamine-nitrosourea compound that damages pancreatic beta-cells through DNA alkylation, oxidative and nitrosative stress, and depletion of cellular energy stores [7,20,22]. In zebrafish, adult protocols have used immersion paradigms as well as intraperitoneal injection. Reported dosing ranges are broad, but this has important biological consequences: lower exposures may produce partial beta-cell injury and more variable glucose elevation with better survival, whereas higher doses or longer exposures generally produce more marked beta-cell loss, more stable hyperglycemia, greater weight loss, and a higher risk of systemic toxicity or mortality [7,22]. STZ models, therefore, usually reflect insulin deficiency or hypoinsulinemia rather than compensatory hyperinsulinemia. STZ combined with a high-fat diet (HFD) induced obesity, elevated blood glucose, and splenomegaly [23].

Alloxan is another beta-cell toxin that acts predominantly through reactive oxygen species and redox cycling, with selective beta-cell vulnerability due in part to glucose transporter-mediated uptake [7]. As with STZ, dose and exposure duration influence the balance between phenotype strength and survival. Lower concentrations may yield weaker or less consistent hyperglycemia, while higher concentrations can intensify beta-cell necrosis and oxidative injury but also increase mortality. Compared with glucose immersion or dietary models, STZ and alloxan are best viewed as acute models of beta-cell injury and insulin deficiency.

Metabolic-Stress Models: Glucose Immersion and Endocrine-Disruptor Exposure

Glucose immersion models expose fish to elevated glucose concentrations in water for days to weeks and are commonly used to generate sustained hyperglycemia, glucose intolerance, and features of insulin resistance [24,25]. In contrast to beta-cell-toxic chemical models, these protocols are more likely to produce early compensatory hyperinsulinemia or altered insulin signaling, especially when exposure is prolonged but not directly beta-cell ablative. They are therefore more aligned with early type 2 diabetes-like metabolic stress than with absolute insulin deficiency. Their main limitations are variability between protocols and the fact that aquatic exposure is not directly analogous to human nutritional intake.

Environmental endocrine disruptors such as bisphenol analogues are better interpreted as diabetogenic or metabolic-disturbance models rather than definitive models of frank diabetes [2]. Depending on the agent, dose, and exposure duration, these protocols may induce glucose intolerance, altered endocrine signaling, insulin resistance-like changes, lipid abnormalities, or oxidative stress, but they do not consistently establish persistent overt diabetes in all settings. Their value lies mainly in studying environmental contributors to metabolic disease risk.

Chemical models are attractive because they are fast, accessible, and flexible. STZ and alloxan are most appropriate when the question involves beta-cell loss, acute hyperglycemia, regeneration, or proof-of-concept screening in insulin-deficient states. Glucose immersion and related metabolic-stress paradigms are more appropriate when the focus is on early insulin resistance-like biology or diabetogenic environmental exposure. Their limitations include protocol variability, dose-toxicity trade-offs, and, for immersion-based paradigms, imperfect physiological correspondence to human disease.

STZ Models

STZ is a glucosamine-nitrosourea compound that selectively destroys pancreatic β-cells through multiple mechanisms, such as glucose transporter-mediated reuptake, DNA alkylation, NAD+ depletion, oxidative stress, and nitrous oxide production [21,23]. Multiple protocols have been developed for STZ-induced diabetes in zebrafish, like the immersion method, where adult zebrafish are exposed for 24-48 hours in STZ dissolved in tank water at 0.3-1.5% (w/v), or the injection method, where STZ is injected intraperitoneally in the zebrafish using citrate buffer as vehicle at a dose of 100-300 mg/kg body weight [8]. The fish show reduced β-cell mass (50-80% reduction), increased β-cell apoptosis, and islet disorganization and fragmentation [23,24]. The STZ model has several applications, like modelling type 1 diabetes and insulin deficiency, screening anti-diabetic compounds and insulin secretagogues, studying β-cell regeneration and repair mechanisms, investigating diabetic complications (retinopathy, nephropathy, neuropathy), and examining oxidative stress and inflammation in diabetes [21,23]. However, this model has a number of limitations, such as variable response, potential toxicity at higher doses, and acute disease development [8].

Alloxan Models

Alloxan is another β-cell toxin that induces diabetes through oxidative stress mechanisms [8]. It works by generating ROS through redox cycling, selective accumulation in β-cells via GLUT2, causing DNA fragmentation and β-cell necrosis, depletes cellular glutathione. Zebrafish are exposed to alloxan at a concentration of 100-400 mg/L in tank water for 24-48 hours. The alloxan acts more rapidly than STZ and has higher mortality rates with more pronounced oxidative stress [8]

Glucose Immersion Models

Glucose immersion models induce hyperglycemia and insulin resistance through chronic exposure to elevated glucose concentrations, more closely mimicking the gradual development of T2DM [24,25]. Shin et al. established optimal protocols for acute hyperglycemia induction [26]. Adult zebrafish were exposed to a glucose concentration of 2-4% (w/v) in tank water with seven to 14 days of exposure for acute models and four to 12 weeks of exposure for chronic models. Normal feeding was maintained. Intine et al. developed high-sugar induced hyperglycemia protocols where glucose concentrations were raised up to 4% for rapid induction with seven to 14 days of exposure for development of stable hyperglycemia [27]. These zebrafishes developed features of hyperglycemia, insulin resistance, compensatory hyperinsulinemia, glucose intolerance, and dyslipidemia.

Dietary induction models

**Figure 3:**
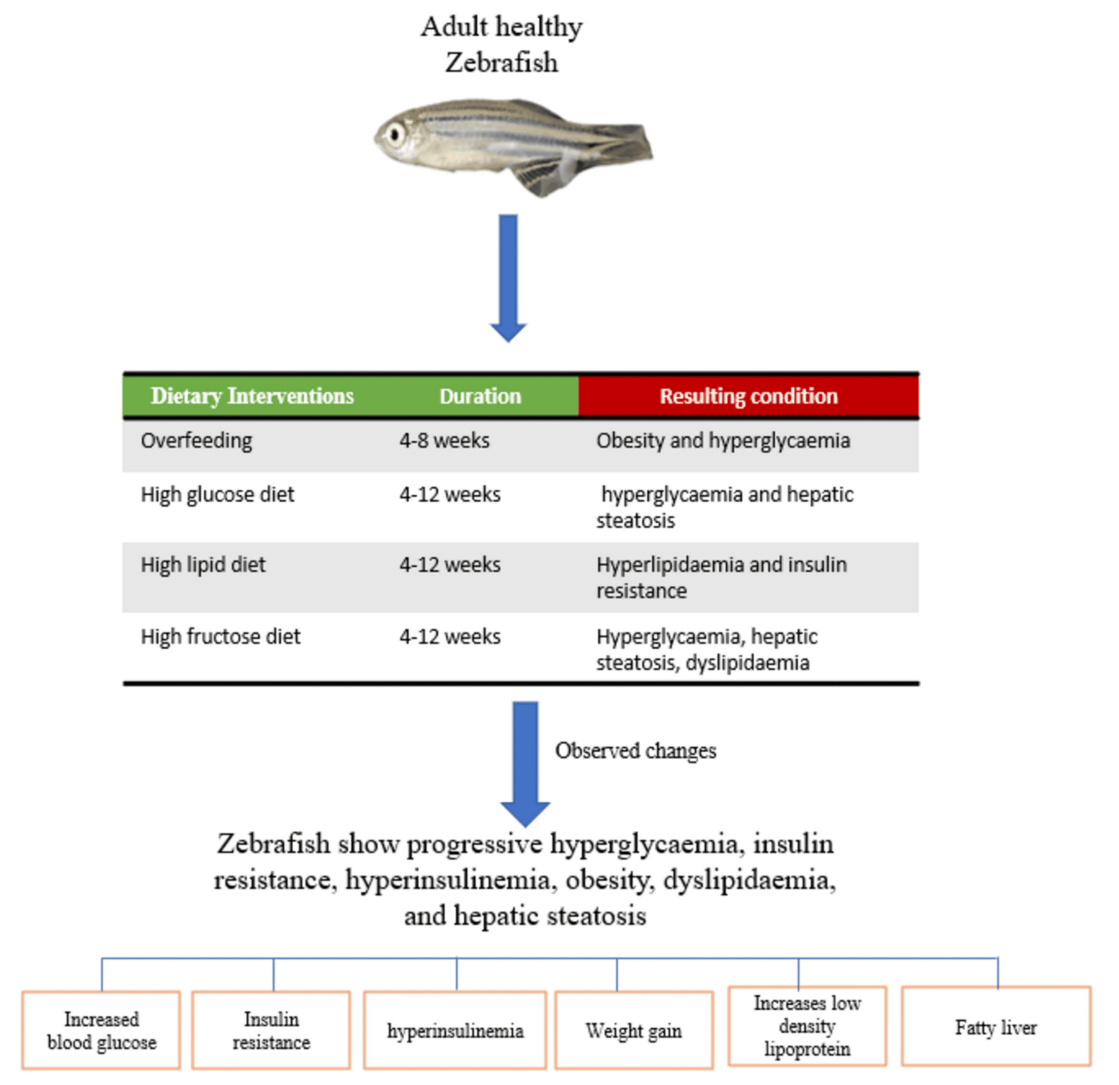
Diet-induced diabetes model in zebrafish Overfeeding and high-fat, high-glucose, or high-lipid diets in adult zebrafish induce diabetes-like metabolic changes, including hyperglycemia, insulin resistance, obesity, dyslipidemia, and hepatic steatosis. The image of the zebrafish is taken from our lab. The rest of the image is made by the authors using Microsoft PowerPoint (Microsoft® Corp., Redmond, WA).

Dietary models most closely mimic human T2DM, which typically develops gradually in response to chronic nutritional excess and obesity [14]. These models are particularly valuable for studying diet-gene interactions, metabolic syndrome, and lifestyle interventions (Figure 3) [3].

High-Fat and Obesogenic Feeding Models

Dietary models most closely mimic the gradual evolution of type 2 diabetes-associated metabolic dysfunction. HFD protocols, including egg-yolk supplemented feed, lipid-enriched Artemia, or other calorie-dense formulations, induce progressive adiposity, glucose intolerance, insulin resistance-like states, dyslipidemia, and hepatic steatosis over several weeks [13,26]. These models are particularly useful when the research question involves obesogenic metabolic stress, diet-gene interaction, gut-liver-pancreas crosstalk, or nutritional interventions.

High-Glucose Feeding, Overfeeding, and Combined Diet-Environment Models

High-glucose diet models and overfeeding paradigms complement high-fat feeding by producing hyperglycemia, enlarged adipose stores, altered inflammatory signaling, and pancreatic dysfunction in a more chronic and behaviorally relevant context [21,25]. Combined high-cholesterol and high-glucose protocols extend this concept further and are useful when vascular injury, steatosis, or mixed metabolic-syndrome phenotypes are of interest [3]. Because these models evolve gradually, they are often better suited than chemical ablation models for studying prevention, reversal, or multi-system metabolic consequences.

Among the available zebrafish paradigms, dietary models are often the most relevant to obesity-associated type 2 diabetes and metabolic syndrome. Their strengths are clinical face validity and the ability to study chronic metabolic stress. Their limitations include longer induction time, greater dependence on husbandry conditions, and potentially higher inter-laboratory variability. They are especially suitable for studies of insulin resistance, steatosis, nutraceuticals, microbiome-linked mechanisms, and longer-term metabolic remodeling.

High-Fat Diet Models

HFD models induce obesity, insulin resistance, and T2DM through chronic lipid overload, mimicking the Western diet pattern associated with diabetes risk in humans [13]. Sarras developed and standardized a novel HFD-induced T2DM model [13]. The diet for this model comprises of 1% egg yolk powder added to standard zebrafish feed, with a three to four times daily feeding frequency over four to eight weeks for adult zebrafish. There is an alternative HFD as well, which comprises enriched brine shrimp (Artemia) with high lipid content or commercial high-fat feeds with Otohime B2 or similar high-calorie formulations [2]. The zebrafish in these models show progressive hyperglycemia, insulin resistance, hyperinsulinemia, obesity, dyslipidemia, and hepatic steatosis [13]. The adult zebrafish show increased visceral adiposity, enlarged liver with pale appearance, abdominal distension, reduced swimming activity, and altered behavior.

High-Glucose Diet Models

High-glucose diets combine glucose supplementation in water with normal or high-calorie feeding [21,26]. Adult zebrafish are exposed to 2-4% glucose in water with normal or increased feeding frequency over a period of four to 12 weeks, which simulates high-sugar diet consumption in humans. High-glucose diet models exhibit hyperglycemia and glucose intolerance, insulin resistance with compensatory hyperinsulinemia, increased adiposity and hepatic steatosis, altered gene expression in glucose and lipid metabolism, oxidative stress and inflammation, pancreatic β-cell dysfunction [21]

Overfeeding Models

Mohammadi et al. characterized overfeeding-induced diabetes in zebrafish [25]. Adult zebrafish are fed three to four times daily ad libitum for four to eight weeks with or without glucose supplementation. Overfeeding models show hyperglycemia, obesity with increased BMI, insulin resistance and glucose intolerance, hepatic steatosis and dyslipidemia, and pancreatic islet dysfunction [21]. Salehpour et al. developed a combined high-cholesterol diet and high-glucose environment model [3], where adult zebrafish are exposed to a high-cholesterol diet (4% cholesterol supplementation), high glucose in water (2%) over eight to 12 weeks. A comparative summary of the models is presented in Table 1.

**Table 1:** Comparative summary of major zebrafish diabetes models This table compares the principal genetic, chemical, and dietary zebrafish diabetes models by induction strategy, dominant phenotype, relative relevance to insulin-deficient versus insulin-resistant states, key strengths, major limitations, and typical research applications. AGE, advanced glycation end products; AKT, protein kinase B; CENP-X, centromere protein X; DIO2, iodothyronine deiodinase 2; GLUT2, glucose transporter 2; glo1, glyoxalase 1; gpr27, G protein-coupled receptor 27; HFD, high-fat diet; INSR, insulin receptor; IRS, insulin receptor substrate; PI3K, phosphoinositide 3-kinase; PDX1, pancreatic and duodenal homeobox 1; slc2a2, solute carrier family 2 member 2; STZ, streptozotocin; T1DM, type 1 diabetes mellitus; T2DM, type 2 diabetes mellitus

Model category	Representative model	Induction method	Principal phenotype in zebrafish	Primary relevance to diabetes type	Key strengths	Major limitations	Typical applications
Genetic	PDX1 mutant	Targeted disruption of PDX1 (for example, frameshift mutation)	Pancreatic hypoplasia, reduced β-cell mass, reduced insulin expression, hyperglycemia, impaired glucose tolerance	Primarily insulin-deficient or β-cell dysfunction model; most relevant to monogenic diabetes and T1DM-like β-cell failure	Mechanistically precise; useful for pancreatic development, β-cell biology, and rescue studies	Monogenic and developmentally weighted; does not capture obesity-associated systemic T2DM complexity	β-cell development, pathway dissection, targeted drug screening
Genetic	GLUT2 (slc2a2) mutant	Targeted disruption of glucose transporter 2	Impaired glucose sensing, altered insulin secretion, hyperglycemia, metabolic dysfunction	β-cell glucose-sensing dysfunction; intermediate relevance between T1DM and T2DM	Direct model of defective glucose sensing and islet physiology	Narrow mechanistic scope; limited representation of whole-body metabolic syndrome	Glucose sensing, insulin secretion, tissue-specific metabolic effects
Genetic	INSR/IRS/PI3K/AKT pathway mutants	Targeted disruption of insulin signaling pathway components	Severe insulin resistance, hyperglycemia despite hyperinsulinemia, altered lipid metabolism, increased adiposity	Primarily T2DM-like insulin resistance	Strong mechanistic model of insulin resistance and signaling defects	Pleiotropic developmental effects may confound metabolic interpretation	Insulin resistance, metabolic signaling, pathway-targeted pharmacology
Genetic	Other targeted models (glo1, Gpr27, DIO2, CENP-X-related models)	Gene knockout, silencing, or modulation	AGE accumulation, oxidative stress, vascular dysfunction, glucose intolerance, altered feeding behavior, impaired metabolism	Model-specific; best viewed as adjunct mechanistic models	Useful for studying discrete mechanisms such as glycation, vascular injury, feeding regulation, or target validation	Less comprehensive diabetic phenotype; heterogeneity limits direct comparison	Complication biology, oxidative stress, candidate target validation
Chemical	Streptozotocin (STZ)	Immersion in STZ solution or intraperitoneal injection	Hyperglycemia with β-cell loss, increased β-cell apoptosis, islet disorganization, insulin deficiency; best interpreted as hypoinsulinemic β-cell injury	Primarily T1DM-like or β-cell ablation model	Rapid, relatively inexpensive, widely used; useful for regeneration and proof-of-concept drug studies	Variable response, dose-dependent toxicity, acute rather than chronic disease course	β-cell ablation, insulin deficiency, regeneration studies, screening anti-diabetic agents
Chemical	Alloxan	Chemical β-cell toxin exposure in tank water	Oxidative β-cell injury, necrosis, hyperglycemia, pronounced oxidative stress, relatively high mortality	Primarily T1DM-like or β-cell injury model	Rapid induction of β-cell damage; useful when oxidative injury is of interest	Higher mortality and toxicity; reproducibility and severity are dose-sensitive	Acute β-cell toxicity, oxidative stress studies, screening of protective compounds
Chemical	Glucose immersion	Chronic exposure to elevated glucose concentrations in tank water	Hyperglycemia, glucose intolerance, insulin resistance, compensatory hyperinsulinemia, dyslipidemia	T2DM-like metabolic dysfunction, especially early insulin-resistant states	Simple, non-invasive, scalable, suitable for high-throughput experiments	Artificial exposure paradigm; models external glucose overload more than full endogenous pathogenesis	Hyperglycemia induction, metabolic screening, early insulin resistance studies
Chemical/environmental	Endocrine disruptor exposure (for example, bisphenol F, bisphenol S)	Exposure to environmental diabetogenic chemicals	Glucose intolerance and metabolic dysfunction resembling aspects of diabetes	Best viewed as diabetogenic-risk or metabolic-disturbance models, not definitive persistent diabetes models	Useful for environmental-metabolic research and exposure biology	Persistent overt diabetes may not be consistently demonstrated; phenotype may be partial rather than full	Environmental risk modelling, endocrine-metabolic disruption studies
Dietary	High-fat diet (HFD)	Lipid-rich feed over several weeks	Progressive hyperglycemia, insulin resistance, hyperinsulinemia, obesity, dyslipidemia, hepatic steatosis	Strong T2DM or metabolic syndrome relevance	Good translational fit for obesity-linked T2DM; captures chronic nutritional excess	Slower induction; phenotype may vary with diet formulation, feeding intensity, and husbandry	Diet-induced insulin resistance, obesity-diabetes interaction, nutraceutical and anti-obesity drug testing
Dietary	High-glucose diet	Glucose supplementation with standard or increased feeding over weeks	Hyperglycemia, glucose intolerance, insulin resistance, compensatory hyperinsulinemia, adiposity, hepatic steatosis	T2DM-like, particularly glucotoxicity-dominant models	Models chronic sugar excess; useful for studying glucotoxicity and metabolic adaptation	Can overlap conceptually with glucose immersion; dietary and exposure effects may be difficult to disentangle	Glucotoxicity, metabolic stress, diet-drug interaction studies
Dietary	Overfeeding	Ad libitum or increased-frequency feeding over several weeks	Obesity, hyperglycemia, insulin resistance, dyslipidemia, and fatty liver	T2DM-like or obesogenic model	Behaviorally and nutritionally relevant; captures caloric excess without gene manipulation	Intake varies across tanks and experiments; standardization can be difficult	Obesity-linked diabetes, lifestyle intervention studies, metabolic syndrome research
Dietary/combined	High-cholesterol plus high-glucose model	Combined cholesterol-rich diet plus glucose-rich water	Mixed metabolic and vascular injury phenotype, with hyperglycemic and dyslipidemic stress	T2DM-like with complication-oriented relevance, especially vascular injury	Useful when metabolic and vascular complications are both of interest	Combined insults reduce mechanistic specificity; harder to attribute phenotype to one pathway	Vascular complications, endothelial injury, complication-focused therapeutic studies

Applications of zebrafish diabetes models

The high-throughput capacity of zebrafish makes them ideal for studies [7,10,19]. The utility of zebrafish diabetes models extends beyond disease induction. Their scalability and imaging accessibility make them especially useful for phenotype-based drug screening, repurposing studies, natural-product evaluation, and dose optimization [6,7,19]. Genetic transparency and the availability of fluorescent reporter lines enable investigators to track beta-cell dynamics, vascular injury, inflammation, and tissue repair in vivo. Zebrafish are also valuable for regeneration research. Because beta-cell recovery can occur after experimental injury, zebrafish provide an unusual vertebrate platform for identifying signals that promote endocrine regeneration and restoration of normoglycemia [22]. This is a major biological advantage for discovery science, but also a reminder that zebrafish do not replicate the limited regenerative capacity of human beta-cells. The study of complications is another important area. Microvascular complications such as retinopathy-like vascular changes, nephropathy-related injury pathways, and neuropathic mechanisms are relatively well developed in zebrafish because the vasculature and relevant tissues can be visualized in living animals [18,20]. Macrovascular and long-latency end-organ consequences can also be explored, but mammalian systems usually remain stronger for modelling the full chronicity and anatomical complexity of human cardiovascular disease.

Benefits of the zebrafish model of diabetes

Diabetes mellitus is a multifactorial metabolic disorder characterized by chronic hyperglycemia arising from defects in insulin secretion, insulin action, or both. The complexity of the disease necessitates the use of animal models that can replicate its diverse pathological features. Historically, mammalian models such as mice, rats, rabbits, and non-human primates have been central to diabetes research due to their physiological similarity to humans. However, the introduction of zebrafish as a model organism has brought a paradigm shift in experimental approaches.

Comparative Physiology and Systemic Metabolism

The most fundamental difference between zebrafish and traditional animal models lies in their physiological organization. Zebrafish are ectothermic, and their metabolic processes are influenced by environmental temperature, while mammalian models are endothermic organisms with tightly regulated internal environments, allowing for stable metabolic homeostasis that closely mirrors human physiology. This stability is critical in studying long-term metabolic disorders such as diabetes, where hormonal regulation, circadian rhythms, and organ interactions play essential roles. It also allows experimental manipulation of metabolic rates under a controlled environment.

Despite these differences, core mechanisms of glucose metabolism are conserved. Zebrafish possess functional insulin, glucagon, and glucose transporter systems, and studies have demonstrated that disruption of insulin signaling leads to hyperglycemia and metabolic imbalance similar to that observed in mammals [27].

Pancreatic Architecture and Beta-Cell Function

In mammalian models, the pancreas is structurally complex, with islets of Langerhans containing multiple endocrine cell types organized in a highly regulated architecture. Beta cells play a central role in insulin secretion, and their dysfunction is a hallmark of diabetes. Zebrafish also possess an endocrine pancreas with insulin-producing beta cells, but the organization is simpler. The principal islet in zebrafish is relatively simple, allowing easier visualization and manipulation.

Functional studies have shown that zebrafish beta cells respond to glucose and regulate systemic glucose levels in a manner comparable to mammals [28]. However, the simplicity of the zebrafish pancreas enables direct observation of beta-cell dynamics in vivo, particularly when using transgenic lines expressing fluorescent markers. This contrasts with mammalian models, where studying beta-cell behavior often requires invasive procedures or post-mortem analysis. Thus, while mammalian pancreatic structure offers closer anatomical similarity to humans, zebrafish provide superior accessibility for studying cellular processes.

Induction of Diabetic States

The induction of diabetes represents another critical area of divergence. In mammalian models, diabetes is commonly induced using chemical agents such as STZ and alloxan, which selectively destroy beta cells, producing a model of type 1 diabetes. Genetic models, including ob/ob and db/db mice, replicate type 2 diabetes through mechanisms involving obesity and insulin resistance. Dietary models further enhance the ability to mimic metabolic syndrome.

Zebrafish models employ both similar and distinct approaches. STZ-induced beta-cell ablation has been successfully demonstrated in zebrafish, indicating conservation of beta-cell susceptibility. However, zebrafish also allow induction of hyperglycemia through immersion in glucose-rich environments, a method not applicable in mammals. Overfeeding and genetic manipulation further enable the modelling of insulin resistance [28].

Regenerative Capacity and Disease Reversibility

A profound difference between zebrafish and mammalian models lies in regenerative biology [29]. Mammalian beta cells exhibit limited regenerative potential, and their loss typically leads to permanent insulin deficiency. This limitation reflects the chronic nature of diabetes in humans but restricts the study of recovery mechanisms. In contrast, zebrafish demonstrate a remarkable ability to regenerate beta cells following injury or ablation. Experimental studies have shown that zebrafish can restore normoglycemia through the regeneration of insulin-producing cells [30].

This regenerative capacity fundamentally alters the interpretation of diabetic models in zebrafish. While mammalian models are well-suited for studying disease progression and chronic complications, zebrafish provide a unique system for investigating mechanisms of tissue regeneration and recovery. The presence of such regenerative pathways introduces biological differences that must be considered when extrapolating findings to human disease, where regeneration is limited.

Genetic Manipulation and Experimental Throughput

The efficiency of genetic manipulation is another major point of comparison. Mammalian models, particularly mice, have well-established genetic engineering techniques, but these processes are time-consuming and resource-intensive. Generating transgenic or knockout mice requires multiple generations and significant infrastructure. Zebrafish, by contrast, allow rapid genetic manipulation through techniques such as CRISPR-Cas9, with results observable within a single [31].

The high fecundity of zebrafish further enhances experimental throughput. Large numbers of embryos can be generated and analyzed simultaneously, enabling large-scale genetic and pharmacological studies. This contrasts with mammalian models, where limited offspring numbers and higher maintenance costs restrict experimental scale. Thus, zebrafish offer a distinct advantage in efficiency, while mammalian systems provide greater depth of physiological relevance.

In Vivo Imaging and Cellular Dynamics

The optical transparency of zebrafish embryos represents a defining feature that distinguishes them from other models. This transparency allows real-time visualization of internal organs, including pancreatic islets and vasculature, without invasive procedures. Researchers can observe beta-cell proliferation, apoptosis, and migration in living organisms, providing dynamic insights into disease processes [32].

In mammalian models, similar observations require advanced imaging techniques or histological analysis, which are often limited by resolution and invasiveness. This difference significantly impacts the type of data that can be obtained. Zebrafish enable continuous, non-invasive observation of disease progression, whereas mammalian models often provide static snapshots. This distinction highlights the complementary nature of these systems in understanding diabetes.

Metabolic Complexity and Disease Modeling

While zebrafish effectively model key aspects of glucose metabolism, the complexity of human diabetes is more comprehensively replicated in mammalian systems. Type 2 diabetes involves interactions between obesity, inflammation, adipokines, and neuroendocrine regulation. Mammalian models capture these interactions more accurately due to their complex organ systems and regulatory networks. Zebrafish can develop obesity-like phenotypes and insulin resistance, but the underlying mechanisms may differ in scale and integration [33].

Compared with mammalian models, zebrafish provide distinct advantages in live imaging, throughput, experimental speed, and genetic manipulability. They allow real-time visualization of pancreatic and vascular biology, efficient creation of targeted mutants, and screening of many compounds or conditions in parallel. These features make zebrafish particularly useful for mechanistic discovery, early translational screening, and regeneration studies [34].

However, zebrafish should be regarded as complementary rather than substitutive models. They are ectothermic, are typically exposed to some interventions through the aquatic environment, and possess stronger regenerative capacity than humans. Their endocrine anatomy and long-term metabolic physiology are informative but not identical to those of mammals. Therefore, while zebrafish can model key aspects of hyperglycemia, beta-cell injury, insulin resistance-like states, and diabetic complications, findings with direct therapeutic or chronic-disease implications often require validation in mammalian systems.

## Conclusions

Zebrafish have become a versatile vertebrate platform for modelling important aspects of diabetes mellitus, ranging from beta-cell injury and insulin deficiency to insulin resistance-like and metabolic dysfunction related to obesity. The range of model systems available for researchers to use (genetic, chemical, and dietary) allows researchers to utilize more flexible resources to answer an array of research-related questions surrounding diabetes pathophysiology, drug discovery, and complications related to diabetes. Genetic models, such as the PDX1 mutant and glucose transporter knockouts, allow for detailed dissection of molecular mechanisms involved in regulating β-cell function and glucose homeostasis. Chemical induction models utilizing a variety of chemical compounds (STZ, alloxan, or glucose immersion) give researchers quick and economical options for creating insulin-deficient and hyperglycemic models. Dietary models, particularly those utilizing an HFD and/or overfeeding methods, most systematically mimic the progressive development of type 2 diabetes in humans, including obesity, insulin resistance, and metabolic syndrome. Although there are some limitations due to physiological differences from mammals, zebrafish models have proven to be excellent tools for replicating many aspects of human diabetes. Continuing developments in technology, including single-cell genomic technologies, CRISPR-based genome-editing technologies, and artificial intelligence, will further extend the range of applications of zebrafish models for diabetes.

A major strength of zebrafish lies in their combination of vertebrate physiology, imaging accessibility, genetic tractability, and experimental throughput. At the same time, their aquatic physiology, protocol heterogeneity, and robust regenerative responses limit direct one-to-one translation to human disease. For that reason, zebrafish should be viewed as highly informative complementary models that can accelerate discovery and sharpen hypothesis selection before downstream validation in mammalian systems.
